# Foveal hypoplasia and characteristics of optical components in patients with familial exudative vitreoretinopathy and retinopathy of prematurity

**DOI:** 10.1038/s41598-022-11455-7

**Published:** 2022-05-11

**Authors:** Pei-Ying Chen, Eugene Yu-Chuan Kang, Kuan-Jen Chen, Xiao Chun Ling, Yin-Hsi Chang, Nan-Kai Wang, Laura Liu, Yen-Po Chen, Yih-Shiou Hwang, Chi-Chun Lai, Wei-Chi Wu

**Affiliations:** 1 Department of Ophthalmology, Linkou Chang Gung Memorial Hospital, No. 5, Fu-Hsin Rd., Taoyuan, 333 Taiwan; 2 College of Medicine, Chang Gung University, Taoyuan, 333 Taiwan; 3 Department of Ophthalmology, Chang Gung Memorial Hospital, Tucheng, 236 Taiwan; 4 Department of Ophthalmology, Chang Gung Memorial Hospital, Keelung, 204 Taiwan; 5 Department of Ophthalmology, Edward S. Harkness Eye Institute, Columbia University Irving Medical Center, Columbia University, New York, 10027 USA; 6 Department of Ophthalmology, Jen-Ai Hospital Dali Branch, Taichung, 412 Taiwan

**Keywords:** Prognostic markers, Retina

## Abstract

There has been limited research regarding the status of foveal hypoplasia and the characteristics of the optical components of the eye in patients with familial exudative vitreoretinopathy (FEVR) and retinopathy of prematurity (ROP). In this retrospective cohort study, patients were classified into five groups: patients with stage 1 and 2 FEVR (FEVR group), patients with ROP who received treatment (treated ROP group), patients with ROP who did not receive treatment (untreated ROP group), patients without ROP who had been born preterm (preterm group), and healthy patients who had been born at term (full-term group). Visual acuity, refractive error, characteristics of the optical components, and features of the fovea were compared. In total, 179 eyes from 100 patients were included. Patients in the FEVR group had the highest degrees of myopia (*p* < 0.001). The axial length of patients in the FEVR group was significantly longer than that of patients in the treated and untreated ROP, preterm, and full-term groups (*p* < 0.001, *p* < 0.001, *p* = 0.001, and *p* = 0.003, respectively). Patients in the FEVR group had a higher proportion of grade 4 foveal hypoplasia and thinner foveae than those in the other groups (*p* < 0.001). Patients with FEVR had significantly greater myopic change than patients with ROP; the significantly longer axial length of the FEVR group might be the reason for the greater myopic change and lesser macular thickness. Patients in the FEVR group had more foveal hypoplasia than those in the other groups.

## Introduction

Familial exudative vitreoretinopathy (FEVR) was first described by Criswick and Schepens in 1969^1^. Patients can present with peripheral avascular retina, disc or macular dragging, retinal folds, retinal neovascularization, vitreous hemorrhage, subretinal exudation, or retinal detachment. The inheritance pattern is variable and can be autosomal dominant, autosomal recessive, or X-linked recessive^2^. This condition can also affect individuals without a family history.

The diagnosis of FEVR is based on the following: (1) a lack of peripheral retinal vascular development in at least one eye; (2) a lack of history of prematurity or preterm birth and a disease time course not consistent with retinopathy of prematurity (ROP); and (3) variable degrees of vitreoretinal traction, subretinal exudation, or retinal neovascularization occurring at any age^3^. FEVR, like ROP, is a disease characterized by abnormal development of retinal vessels. Both diseases share several similar clinical features such as peripheral avascular retina, dragging of the retinal vessels, abnormalities in retinal vessel branching, retinal neovascularization, and retinal detachment.

The main difference between FEVR and ROP is a history of prematurity. However, there is a subgroup of FEVR patients who are born prematurely; this condition is known as “ROPER”^4^. Therefore, it might sometimes be difficult to differentiate the two diseases based on history alone. To address this issue, the current study aimed to examine subtle differences between FEVR and ROP. Additionally, the characteristics of children born at term and preterm were also investigated. In this retrospective study, several parameters, such as refractive errors, optical components, and foveal development, were analyzed and compared to determine the similarities and differences between patients with FEVR and those with ROP.

## Materials and methods

### Study design

This retrospective study included patients with FEVR, ROP, and prematurity without ROP in Chang Gung Memorial Hospital between 2010 and 2018. We also recruited an age-matched group of healthy, full-term patients from our clinic during this time period. Institutional Review Board (IRB) approval was obtained from Chang Gung Medical Foundation (IRB number 201901753B0, 201900571B0). Informed consent was waived by the IRB due to retrospective design and the use of deidentified patient information. This study adhered to the tenets of the Declaration of Helsinki. The clinical staging of FEVR was classified according to the staging system previously published^5^. Stage 1 was defined as avascular peripheral retina or anomalous intraretinal vascularization, and stage 2 was defined as avascular peripheral retina with extraretinal vascularization.

Patients were excluded if they had retrolental fibroplasia, retinal detachment, retinal folds, or epiretinal membranes or if they had received any intraocular surgery other than laser photocoagulation and intravitreal injection. Patients with other ocular diseases such as glaucoma, uveitis, and cataracts were excluded as well. Enrolled patients were classified into five groups: patients with stage 1 or stage 2 FEVR (FEVR group), ROP patients who had been treated with laser therapy or intravitreal injection (treated ROP group), ROP patients who had not received any treatment (untreated ROP group), patients with a history of preterm birth without ROP (preterm group), and healthy patients born at term (full-term group). Treatment was indicated when the severity reached type 1 ROP as defined by Early Treatment for Retinopathy of Prematurity (ETROP)^6^. Patients with type 2 or mild ROP were monitored closely until the complete regression of ROP.

Basic characteristics such as gestational age (GA), birth weight (BW), sex, and age at the time of examination were obtained from medical charts. Data including refractive error, uncorrected and corrected visual acuity (VA), spherical and cylindrical power, keratometry, anterior chamber depth, and axial length were recorded and compared between groups.

### Measures

Automatic cycloplegic refraction was performed by an automatic keratorefractometer (KR-800, Topcon, Tokyo, Japan) followed by manual refraction to achieve optimal results. Optical component characteristics, including axial length and anterior chamber depth, were measured with IOL-Master (Carl Zeiss Meditec, Jena, Germany) and compared between groups of patients. Snellen VA was converted to the logarithm of the minimum angle of resolution (logMAR) VA for statistical analysis.

The structural grading of foveal hypoplasia was defined in a previous study^7^. The OCT scan was performed on an SD-OCT device (Spectralis, Heidelberg Engineering, Heidelberg, Germany). To avoid bias, serial SD-OCT scans over the macular area of each patient were carefully checked to determine the accurate localization of the fovea. Then, the foveal volume, foveal thickness, parafoveal thickness, and perifoveal thickness were automatically calculated by the OCT system. The grading of foveal development was recorded according to the classification system and compared between groups. Interpretations of OCT scans were made by two retina ophthalmologists with more than 15 years of experience (KJC and YSH) and reviewed by a third retinal ophthalmologist (WCW). If there were discrepancies among graders or segmentation errors, the OCT image was checked by all graders and corrected manually. Poor-quality scans, which were defined as images with (1) a signal quality of less than 20 dB or (2) segmentation errors or artifacts, were excluded.

### Statistical analysis

Categorical variables were compared by using chi-square tests. Numerical variables between the study groups were compared by using generalized estimating equations based on a previous publication^8^, with adjustments for correlation between the two eyes of each subject. Intergroup comparisons were made using post hoc analyses. Statistical significance was considered as a *p-*value less than 0.05. All statistical analyses were performed using Statistical Product and Service Solutions (SPSS, Version 22.0. Armonk, NY).

## Results

### Demographics

In total, 179 eyes of 100 patients were included in this cohort, and the mean age of the studied participants was 7.2 years. All of the patients were of Asian descent. Demographic data on the five groups of patients are shown in Table 1. In the FEVR group, 24 eyes were classified as stage 1 FEVR, and 10 eyes were classified as stage 2 FEVR. The mean GA and BW of patients in the FEVR group were 38.2 ± 2.1 weeks and 3074.1 ± 469.8 g, respectively, which were comparable to those of patients in the full-term group. The mean GA and BW of patients in the ROP and preterm groups were significantly lower than those of the other groups (*p* < 0.001). The mean age at the time of examination was not significantly different (*p* = 0.250) between each group. There was no significant difference in sex distribution among groups (*p* = 0.380).

**Table 1:** Patient demographics in the different study groups.

	FEVR	Treated ROP	Untreated ROP	Preterm	Full-term	*p* value
No. of eyes (patients)	34 (24)	36 (20)	32 (16)	38 (20)	39 (20)	
Age, yrs	7.9 ± 3.9	7.4 ± 2.4	5.8 ± 1.3	7.1 ± 2.4	7.0 ± 2.7	0.25
GA, wks	38.2 ± 2.1	26.3 ± 2.3	27.8 ± 2.2	32.3 ± 3.0	38.8 ± 1.2	< 0.001
BW, g	3074.1 ± 469.8	819.6 ± 203.6	916.4 ± 355.3	1600.1 ± 501.8	3036.6 ± 477.7	< 0.001
**Sex, no. of patients (%)**						0.38
Male	14 (58%)	9 (45%)	8 (50%)	12 (60%)	15 (75%)	
Female	10 (42%)	11 (55%)	8 (50%)	8 (40%)	5 (25%)	
**Treatment, no. of eyes (patients)**						
Laser	20 (16)	12 (7)	–	–	–	
IVI of anti-VEGF	–	20 (11)	–	–	–	
Laser + IVI of anti-VEGF	–	4 (2)	–	–	–	

FEVR: familial exudative vitreoretinopathy; ROP: retinopathy of prematurity; GA: gestational age; BW: birth weight; IVI: intravitreal injection; VEGF: vascular endothelial growth factor.

### Refractive error and optical components

The refractive error and optical component data are shown in Table 2. When compared with the preterm and full-term groups, the treated ROP and untreated ROP groups had higher degrees of myopia, higher astigmatism, and steeper corneal curvature but a similar axial length. The uncorrected and corrected logMAR VAs of the FEVR group were significantly worse than those of the other groups (*p* < 0.001). Patients in the FEVR group had significantly higher degrees of myopia than patients in the ROP with and without treatment, preterm, and full-term groups (*p* = 0.002, *p* < 0.001, *p* < 0.001, and *p* < 0.001, respectively). The axial length was significantly longer in patients in the FEVR group than in the other groups (*p* < 0.001, *p* < 0.001, *p* = 0.001, and *p* = 0.003, respectively). There was no significant difference in corneal curvature or anterior chamber depth between the FEVR group and the other groups. When the untreated ROP group was compared with the full-term group, the former was noted to have steeper corneal curvature (*p* = 0.002) but comparable anterior chamber depth and axial length.

**Table 2:** Refractive error and optical-component features of patients.

	FEVR (1)	Treated ROP (2)	Untreated ROP (3)	Preterm (4)	Full-term (5)	*p* value	Post hoc^a^
Uncorrected logMAR VA	1.2 ± 0.7	0.5 ± 0.5	0.2 ± 0.3	0.1 ± 0.2	0.2 ± 0.3	< 0.001	1-2, 1-3, 1-4, 1-5, 2-4, 2-5
Corrected logMAR VA	0.5 ± 0.7	0.07 ± 0.2	0.02 ± 0.1	0.03 ± 0.1	0.03 ± 0.1	< 0.001	1-2, 1-3, 1-4, 1-5
Spherical power, D	− 6.2 ± 6.8	− 1.4 ± 4.6	0.3 ± 1.4	0.4 ± 2.9	0.5 ± 0.9	< 0.001	1-2, 1-3, 1-4, 1-5
Cylindrical power, D	− 1.6 ± 1.3	− 1.6 ± 1.6	− 1.1 ± 1.6	− 0.5 ± 0.5	− 0.4 ± 0.7	< 0.001	1-4, 1-5, 2-4, 2-5, 3-5
Spherical equivalent, D	− 7.0 ± 7.0	− 2.2 ± 4.8	− 0.3 ± 1.6	0.2 ± 2.9	0.3 ± 0.9	< 0.001	1-2, 1-3, 1-4, 1-5, 2-5
**Keratometry**							
K1	42.7 ± 1.9	44.2 ± 1.9	43.8 ± 1.9	43.0 ± 2.2	42.3 ± 1.6	0.006	1-2, 2-5, 3-5
K2	44.7 ± 1.9	46.1 ± 2.1	45.6 ± 1.7	44.0 ± 2.4	43.7 ± 1.7	< 0.001	2-4, 2-5, 3-4, 3-5
K1-K2 average	43.7 ± 1.8	45.2 ± 1.9	44.7 ± 1.7	43.5 ± 2.3	43.0 ± 1.6	0.001	2-4, 2-5, 3-5
Anterior chamber depth, mm	3.2 ± 0.4	3.2 ± 0.3	3.1 ± 0.4	3.2 ± 0.4	3.3 ± 0.2	0.067	
Axial length, mm	24.4 ± 2.6	22.4 ± 1.0	22.3 ± 1.1	22.7 ± 1.0	22.9 ± 0.7	0.001	1-2, 1-3, 1-4, 1-5

FEVR: familial exudative vitreoretinopathy; ROP: retinopathy of prematurity; VA: visual acuity; D: diopters.

^a^Intergroup comparisons were made using the post hoc comparisons from significant generalized estimating equations; the pairs of groups listed (e.g., 1-2) are significantly different. Pairs not shown are not significantly different.

### Foveal structure

The grade of foveal hypoplasia in the five groups is shown in Table 3. Patients in the FEVR group had a higher proportion of foveal hypoplasia than patients in the other groups. The number (and percentage) of patients with grade 4 foveal hypoplasia was 8 (23.5%) in the FEVR group, 1 (2.8%) in the treated ROP group, 0 (0%) in the untreated ROP group, 0 (0%) in the preterm group, and 0 (0%) in the full-term group (*p* < 0.001). Two eyes in the FEVR group had unjudgeable OCT images and were excluded from the analysis of foveal structures.

**Table 3:** Foveal hypoplasia in each group.

Group	FEVR (1)	Treated ROP (2)	Untreated ROP (3)	Preterm (4)	Full-term (5)
Absence of foveal hypoplasia	14 (41.2%)	20 (55.6%)	27 (84.4%)	38 (100%)	39 (100%)
Grade 1 (absence of the extrusion of plexiform layers)	9 (26.5%)	14 (38.9%)	5 (15.6%)	0 (0%)	0 (0%)
Grade 2 (absence of the fovea pit)	1 (2.9%)	1 (2.8%)	0 (0%)	0 (0%)	0 (0%)
Grade 3 (absence of OS lengthening)	0 (0%)	0 (0%)	0 (0%)	0 (0%)	0 (0%)
Grade 4 (absence of ONL widening)	8 (23.5%)	1(2.8%)	0 (0%)	0 (0%)	0 (0%)
No OCT data^a^	2	0	0	0	0

FEVR: familial exudative vitreoretinopathy; ROP: retinopathy of prematurity; OS: outer segment; ONL: outer nuclear layer; OCT: optical coherence tomography.

*p* value: < 0.001.

^a^OCT scans that could not be judged by ophthalmologists.

The volume and thickness of the fovea in each group are shown in Table 4. The FEVR group had the lowest foveal volume (*p* < 0.001). The thickness of the fovea was significantly thinner in the FEVR group than in the treated ROP (*p* < 0.001), untreated ROP (*p* = 0.002), and preterm (*p* = 0.01) groups. The parafoveal and perifoveal areas of the retina were thinner in the FEVR group than in any other group (*p* < 0.001).

**Table 4:** Macular structures in each group.

	FEVR (1)	Treated ROP (2)	Untreated ROP (3)	Preterm (4)	Full-term (5)	*p* value	Post hoc^a^
Volume, mm^3^	7.6 ± 1.2	8. 9 ± 0.7	8.4 ± 0.5	8.8 ± 0.8	8.8 ± 0.5	< 0.001	1-2, 1-3, 1-4, 1-5, 2-3, 3-5
Foveal thickness, µm	250.9 ± 40.6	291.0 ± 34.8	281.4 ± 25.1	276.9 ± 41.7	257.9 ± 46.4	0.001	1-2, 1-3, 1-4, 2-5, 3-5
**Parafoveal thickness, µm**	284.3 ± 43.5	337.8 ± 36.2	323.2 ± 17.0	335.1 ± 23.5	337.2 ± 19.1	< 0.001	1-2, 1-3, 1-4, 1-5, 2-3, 3-4, 3-5
Temporal	275.2 ± 50.6	325.1 ± 38.0	318.7 ± 30.3	325.3 ± 23.6	327.9 ± 19.4	< 0.001	1-2, 1-3, 1-4, 1-5
Nasal	292.8 ± 42.7	339.9 ± 38.2	328.7 ± 19.2	337.9 ± 19.1	342.6 ± 23.4	< 0.001	1-2, 1-3, 1-4, 1-5, 3-5
Superior	285.3 ± 48.4	347.0 ± 43.1	325.5 ± 11.6	341.5 ± 39.7	339.8 ± 12.4	< 0.001	1-2, 1-3, 1-4, 1-5, 2-3, 3-4, 3-5
Inferior	284.0 ± 40.9	338.9 ± 59.9	319.8 ± 21.7	335.7 ± 36.9	338.4 ± 41.4	< 0.001	1-2, 1-3, 1-4, 1-5, 3-5
**Perifoveal thickness, µm**	262.2 ± 45.3	306.1 ± 26.3	294.3 ± 21.7	306.4 ± 30.9	307.3 ± 16.9	< 0.001	1-2, 1-3, 1-4, 1-5, 3-5
Temporal	248.6 ± 47.6	300.5 ± 31.4	279.7 ± 26.9	294.0 ± 37.3	297.4 ± 17.8	< 0.001	1-2, 1-3, 1-4, 1-5, 2-3, 3-5
Nasal	285.2 ± 51.0	321.3 ± 30.9	308.5 ± 20.3	317.5 ± 19.8	324.5 ± 23.5	0.007	1-2, 1-4, 1-5, 3-5
Superior	260.0 ± 51.1	309.4 ± 30.7	302.8 ± 34.7	321.7 ± 76.9	307.7 ± 15.2	< 0.001	1-2, 1-3, 1-4, 1-5
Inferior	255.0 ± 42.6	293.4 ± 27.2	285.9 ± 31.9	292.5 ± 26.8	299.5 ± 28.1	< 0.001	1-2, 1-3, 1-4, 1-5

FEVR: familial exudative vitreoretinopathy; ROP: retinopathy of prematurity.

^a^Intergroup comparisons were made using the post hoc comparisons from significant generalized estimating equations; the pairs of groups listed (e.g., 1-2) are significantly different. Pairs not shown are not significantly different.

### Comparison of stage 1 and stage 2 FEVR

Table 5 shows parameters including refractive error, optical component characteristics, and macular structure in patients with stage 1 and stage 2 FEVR. In an analysis of 22 eyes with stage 1 FEVR and 10 eyes with stage 2 FEVR, anterior chamber depth and foveal thickness were significantly different between stage 1 and stage 2. Regarding anterior chamber depth, patients with stage 1 FEVR had lesser depth than those with stage 2 FEVR (*p* = 0.002). Furthermore, the foveal thickness was significantly thicker in stage 2 than in stage 1 (*p* = 0.004).

**Table 5:** Parameters of eyes with familial exudative vitreoretinopathy.

	Stage 1 (n = 22)	Stage 2 (n = 10)	*p* value
Uncorrected logMAR VA	1.3 ± 0.7	1.2 ± 0.8	0.741
Corrected logMAR VA	0.7 ± 0.7	0.3 ± 0.4	0.140
Spherical power, D	− 7.0 ± 6.9	− 4.4 ± 6.7	0.315
Cylindrical power, D	− 1.6 ± 1.4	− 1.5 ± 0.9	0.866
Spherical equivalent, D	− 7.8 ± 7.2	− 5.1 ± 6.7	0.322
**Keratometry**			
K1	42.9 ± 2.1	42.4 ± 1.4	0.525
K2	44.9 ± 2.0	44.4 ± 1.6	0.443
K1-K2 average	43.9 ± 1.9	43.4 ± 1.5	0.467
Anterior chamber depth, mm	3.1 ± 0.4	3.6 ± 0.4	0.002
Axial length, mm	24.1 ± 2.1	25.2 ± 3.6	0.394
Macular structures in OCT			
**Foveal hypoplasia**			
Absence of foveal hypoplasia	10 (45.5%)	4 (40.0%)	0.881
Grade 1 (absence of the extrusion of plexiform layers)	6 (27.3%)	3 (30.0%)	
Grade 2 (absence of the fovea pit)	0 (0%)	0 (0%)	
Grade 3 (absence of OS lengthening)	1 (4.5%)	0 (0%)	
Grade 4 (absence of ONL widening)	5 (22.7%)	3 (30.0%)	
Macular volume, mm^3^	7.3 ± 0.9	8.0 ± 1.6	0.082
Foveal thickness, µm	239.9 ± 40.8	274.3 ± 30.3	0.004
Parafoveal thickness, µm	277.6 ± 42.9	300.3 ± 43.2	0.055
Perifoveal thickness, µm	254.2 ± 30.0	281.4 ± 68.6	0.088

VA: visual acuity; D: diopters; OCT: optical coherence tomography; OS: outer segment; ONL: outer nuclear layer.

## Discussion

In this study, we found that the FEVR group had significantly poorer uncorrected and corrected VA than the other groups. Uncorrected and corrected VA became progressively worse from the full-term group to the FEVR group. While myopia was common in patients with ROP, a greater myopic change was noted in patients with FEVR. There was a gradual progression of refractive error from the full-term group to the FEVR group. The axial length of the FEVR group was significantly longer than that of any other group. Patients in the FEVR group had an increased prevalence of foveal hypoplasia and thinner fovea. From these observations, there are some subtle but noteworthy differences between patients with FEVR and patients with ROP.

### Optical components

A previous study noted an association between myopia development and FEVR^9^. FEVR patients presented with excessive myopia and amblyopia since early childhood. In addition, all patients had excessive myopia, ranging from − 3.5 to − 16.75 diopters reported from the study. High refractive error in ROP patients was related to the optical components in the anterior segment, such as a shallow anterior chamber depth and steep cornea curvature^10,11^. In patients with FEVR, however, the long axial length, rather than any abnormality in the anterior segment, was responsible for myopia. This study suggests that the mechanisms of myopia are different in FEVR and ROP.

### Macular structure

Macular microvasculature abnormalities have been reported in both FEVR and ROP patients. A previous study revealed that some patients with stage 1 and 2 FEVR had persistence of inner retinal layers in the fovea, which is analogous to mild foveal hypoplasia^12^. A significantly small foveal avascular zone (FAZ) and decreased vascular density of the parafoveal area were discovered in a case series including 41 eyes^13^. Decreased density and disorganization of the deep vascular complex were revealed by OCT angiography in a series of 11 eyes^14^. In patients with threshold ROP, an abnormal foveal contour and retention of the inner retinal layers were noted^11^. The mean FAZ area was significantly smaller and the mean central retina thickness was significantly thicker in patients with ROP^15^. In this study, patients with FEVR had a higher proportion of grade 4 foveal hypoplasia than those with ROP who did or did not receive treatment. In addition, in this study, the foveal, parafoveal, and perifoveal areas were thinner in FEVR patients than in ROP and preterm patients. Although foveal hypoplasia has been reported to be associated with increased foveal thickness^7^, the foveal thickness of FEVR patients was similar to that of full-term controls. A possible explanation of the difference in foveal thickness among the FEVR, ROP, and full-term groups is the significantly longer axial length in the FEVR group, which may cause not only greater myopia but also lesser retinal thickness^16^.

### Difference between stage 1 and 2 FEVR

In the FEVR group, the foveal thickness was significantly greater in stage 2 patients than in stage 1 patients. A similar finding was observed in a previous study reported by Yonekawa et al.^12^, which demonstrated that the mean foveal thickness values in patients with stage 1 FEVR and stage 2 FEVR were 271 µm and 358 µm, respectively. In this study, since axial length was comparable between patients with stage 1 and stage 2 FEVR, greater maldevelopment of the fovea in stage 2 disease might be the reason for the increased foveal thickness of stage 2 patients compared with stage 1 patients. As for anterior chamber depth, this measure has been reported to be associated with global retinal nerve fiber layer thickness, age, spherical equivalent, and axial length^6^. However, in our study, stage 2 FEVR was significantly associated with a deeper anterior chamber than stage 1 FEVR, while spherical equivalent and axial length showed no significant difference. The development of the anterior chamber in the different stages of FEVR may need further investigation.

### Clinical characteristics of FEVR and ROP

**Figure 1:**
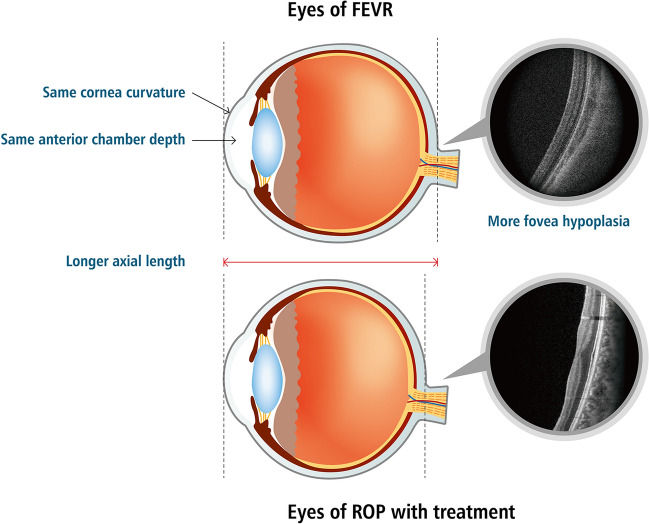
Comparison of optical-component characteristics and fovea status between patients with familial exudative vitreoretinopathy (FEVR) and patients with retinopathy of prematurity (ROP) who had received treatment.

In the present study, patients with FEVR had a higher proportion of grade 4 foveal hypoplasia than those with ROP. The axial length of patients with FEVR was significantly greater than that of patients with ROP. A comparison of optical components and foveal status between patients with FEVR and patients with ROP who had received treatment is shown in Fig. 1.

**Figure 2:**
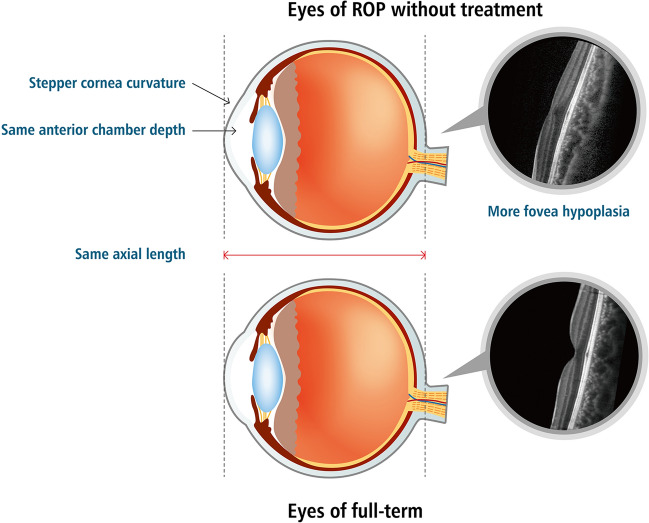
Comparison of optical-component characteristics and fovea status between patients with retinopathy of prematurity (ROP) who had not received treatment and full-term patients.

In this study, treated ROP patients tended to have poorer uncorrected and corrected VA, greater degrees of myopia, higher astigmatism, and steeper corneal curvature than untreated ROP patients, preterm patients, or full-term patients. However, there was no significant difference in axial length in the ROP group compared to the. This result was compatible with the characteristics of patients with threshold ROP in a previous study^11^, in which no significant difference was found in uncorrected and corrected VA, myopia, anterior chamber depth, or axial length between ROP patients who had not received treatment and full-term patients. Corneal curvature was significantly steeper in ROP patients who had not received treatment than in full-term patients. A comparison of optical components and foveal status between patients with ROP who had not received treatment and full-term patients is shown in Fig. 2.

FEVR is thought to occur in full-term patients with familial history and genetic predisposition, while ROP is found in premature infants and has a more predictable course of progression. In contrast to ROP, FEVR tends to recur in late childhood, adolescence, or even later in age with neovascularization, vitreous hemorrhage, or retinal detachment. Regarding the findings of fluorescein angiography (FA), abnormal vessel branching patterns, neovascularization, and leakage are observed in ROP patients^17^. In FEVR patients, FA shows branching vessels with bulb-like telangiectatic endings, leakage, and venous-venous looping^18^. However, some preterm patients exhibit a disease course and clinical findings that are consistent with FEVR. A new classification of ROPER (ROP vs. FEVR) was therefore proposed^4^. In the genetic field, both FEVR and ROP were previously found to be associated with mutations in genes in Wnt signaling^19^. Several FEVR-associated mutations have also been found in patients with advanced ROP. Mutations in the FZD4 gene were observed in 7.5% of patients with severe ROP^20^. Another study revealed that 13% of patients with advanced ROP carry mutations in FZD4 or LRP5^21^. The overlapping clinical presentations and genetic similarities suggest a clinical spectrum between ROP and FEVR.

### Limitations

This study has some limitations. First, the retrospective design of this study may render bias inevitable. Second, the small case number in each group could have affected statistical significance. Third, this study included FEVR patients with only stage 1 or stage 2 disease because these patients had pathologies that resembled those of type 1 ROP (needing treatment) or milder ROP (not needing treatment). The characteristics of more severe FEVR may need further study. Fourth, there was a lack of genetic results in our study, and the association of the clinical features with genetic abnormalities could not be determined. In addition, the study did not include patients with ROPER because FA and genetic testing were unavailable. The diagnosis of ROPER, which was proposed in 2016^4^, was also not well established in the study cohort during their infancy. However, this is the first study to make direct comparisons between patients with FEVR and those with ROP. The fine-grained differences between these conditions could offer additional understanding for future ROPER studies and improve the clinical distinction between these two groups of patients when other clinical features are ambiguous.

In conclusion, this retrospective study revealed poorer visual function and different optical components in FEVR patients than in ROP patients. In FEVR patients, the disease caused a more significant impact on uncorrected and corrected VA and foveal development than in ROP patients. There was less anterior segment change, which was attributed to the high degrees of myopia in ROP patients. Instead, a longer axial length correlated with high refractive error. Uncorrected and corrected VA, refractive error, and axial length tended to progress from the full-term group to the FEVR group. These findings might suggest a disease spectrum from mild to severe. Further analysis of the disease mechanism needs to be conducted in the future.
